# Differential Immune Profiles in Two Pandemic Influenza A(H1N1)pdm09 Virus Waves at Pandemic Epicenter

**DOI:** 10.1016/j.arcmed.2015.12.003

**Published:** 2015-11

**Authors:** Lourdes Arriaga-Pizano, Eduardo Ferat-Osorio, Gabriela Rodríguez-Abrego, Ismael Mancilla-Herrera, Esteban Domínguez-Cerezo, Nuriban Valero-Pacheco, Marisol Pérez-Toledo, Fernando Lozano-Patiño, Fernando Laredo-Sánchez, José Malagón-Rangel, Haiko Nellen-Hummel, César González-Bonilla, Gabriel Arteaga-Troncoso, Arturo Cérbulo-Vázquez, Rodolfo Pastelin-Palacios, Paul Klenerman, Armando Isibasi, Constantino López-Macías

**Affiliations:** a Medical Research Unit in Immunochemistry, Specialties Hospital, National Medical Center Siglo XXI, IMSS, Mexico City, Mexico; b Gastrointestinal Surgery Service, Specialties Hospital, National Medical Center Siglo XXI, IMSS, Mexico City, Mexico; c Clinical Epidemiology Service, Regional Hospital No, 1, IMSS, Mexico; d Infectology and Immunology department, National Institute of Perinatology, SSA, Mexico City, Mexico; h Cellular Biology department, National Institute of Perinatology, SSA, Mexico City, Mexico; e Graduate Program on Immunology, ENCB-IPN, Mexico City, Mexico; f Internal Medicine Service, Specialties Hospital of the National Medical Center Siglo XXI, IMSS, Mexico City, Mexico; g Unit for Epidemiological Surveillance, National Medical Center La Raza, IMSS, Mexico City, Mexico; i Education and Research Division, Woman's Hospital, Health Ministry, Mexico City, Mexico; j Chemistry Faculty, National Autonomous University of Mexico (UNAM), Mexico City, Mexico; k Oxford Biomedical Research Centre and Oxford Martin School, Nuffield Department of Medicine, University of Oxford, Oxford, UK; l Visiting Professor of Immunology, Nuffield Department of Medicine, University of Oxford, Oxford, UK

**Keywords:** Immune profiles, Pandemic A(H1N1)pdm2009 influenza, Pandemic waves

## Abstract

**Background and Aims:**

Severe influenza A(H1N1)pdm2009 virus infection cases are characterized by sustained immune activation during influenza pandemics. Seasonal flu data suggest that immune mediators could be modified by wave-related changes. Our aim was to determine the behavior of soluble and cell-related mediators in two waves at the epicenter of the 2009 influenza pandemic.

**Methods:**

Leukocyte surface activation markers were studied in serum from peripheral blood samples, collected from the 1^st^ (April–May, 2009) and 2^nd^ (October 2009–February 2010) pandemic waves. Patients with confirmed influenza A(H1N1)pdm2009 virus infection (H1N1), influenza-like illness (ILI) or healthy donors (H) were analyzed.

**Results:**

Serum IL-6, IL-4 and IL-10 levels were elevated in H1N1 patients from the 2^nd^ pandemic wave. Additionally, the frequency of helper and cytotoxic T cells was reduced during the 1^st^ wave, whereas CD69 expression in helper T cells was increased in the 2^nd^ wave for both H1N1 and ILI patients. In contrast, CD62L expression in granulocytes from the ILI group was increased in both waves but in monocytes only in the 2^nd^ wave. Triggering Receptor Expressed on Myeloid cells (TREM)-1 expression was elevated only in H1N1 patients at the 1^st^ wave.

**Conclusions:**

Our results show that during the 2009 influenza pandemic a T cell activation phenotype is observed in a wave-dependent fashion, with an expanded activation in the 2^nd^ wave, compared to the 1^st^ wave. Conversely, granulocyte and monocyte activation is infection-dependent. This evidence collected at the pandemic epicenter in 2009 could help us understand the differences in the underlying cellular mechanisms that drive the wave-related immune profile behaviors that occur against influenza viruses during pandemics.

## Introduction

Influenza A(H1N1)pdm09 virus infection resulted in high morbidity and mortality rates in young adults during the Mexican outbreak 1, 2. Some underlying conditions (3) or simultaneous contact with infectious foci (4) were related to influenza severity. Acute manifestations in young-immunocompetent subjects resembled infections by highly pathogenic pandemic influenza viruses, such as the H5N1 avian influenza and the 1918 H1N1 virus (2). The immunological profiles of previous pandemics (e.g., 1918, 1957 and 1968) showed hypercytokinemia and exacerbated leukocyte activation 5, 6, making it relevant to characterize the inflammatory response to the influenza A(H1N1)pdm09 virus (7).

Multiple waves during influenza pandemics have been observed 8, 9, 10, and mathematical models show that viral mutations (11), social interventions 12, 13, 14, 15 and ratio of immunocompromised individuals 16, 17, 18 determine the potential and magnitude of subsequent waves. These theoretical models are essential for analyzing multiple influenza outbreak waves and pandemic planning (19). In addition, seasonal influenza has shown different immune behaviors among waves 20, 21. However, no evidence has been reported regarding the wave-related changes for immune soluble and cellular markers in the first influenza pandemic of the 21^st^ century.

In addition to soluble mediators, surface cellular markers such as CD69 (an early lymphocyte activation marker), lung homing related CD62L, Triggering Receptor Expressed on Myeloid cells (TREM)-1 and HLA-DR have been useful for inflammation analysis in influenza infections (22), inflammatory systemic syndrome (23) and hypersensitivity states (24).

Furthermore, multi-wave epidemics such as in Mexico 2009 offer opportunities to test the immune nature of short-term shifts in humoral and cellular responses. We report the immunological profiles (cytokine and chemokine levels, proportion of circulating leukocytes and activation phenotype, based on surface expression markers) in RT-PCR verified patients for influenza A(H1N1)pdm2009 virus (H1N1), influenza-like illness (ILI) and healthy donors (H) during two infection waves in the epicenter of the Mexican outbreak.

## Materials and Methods

### Patients and Sample Collection

Our study was conducted by the Medical Research Unit in Immunochemistry (UIMIQ) and approved by the Ethics and Research Committee of the National Medical Center Siglo XXI, IMSS, Mexico City (Research project: CNIC 2010-785-002). Mexico City patients from the 1^st^ (April–May 2009) and 2^nd^ (October 2009–February 2010) pandemic waves were enrolled.

Sixty-two patients from the Specialties Hospital of the National Medical Center Siglo XXI, IMSS or the Regional Hospital “Dr. Carlos MacGregor”, and 12 healthy volunteers were analyzed. All subjects were included after informed consent had been read and signed according to guidelines established by the local ethics committee. Study groups were classified into the following: a) influenza-like illness (ILI) patients from the 1^st^ (*n* = 10) and 2^nd^ (*n* = 20) waves; b) confirmed influenza A(H1N1)pdm09 virus-infected (H1N1) patients from the 1^st^ (*n* = 9) and 2^nd^ (*n* = 23) waves; or c) healthy volunteers (H, *n* = 12). None of the patients from the 2^nd^ wave was recruited in the 1^st^ one. ILI was defined as a combination of cough, headache, and fever with one or more of the following symptoms: sore throat, rhinorrhea, arthralgia, myalgia, prostration, thoracic pain, abdominal pain, nasal congestion, diarrhea and irritability, as previously described (25). All patients were tested for influenza A(H1N1)pdm09 viral infection using specific real-time reverse transcription–polymerase chain reaction (rRT-PCR) according to the CDC protocol of real-time RT-PCR for swine influenza (H1N1) (26). The analysis was performed at the Unit for Epidemiological Surveillance, National Medical Centre “La Raza”, IMSS, Mexico City.

All participants were clinically evaluated at the time of their first admission in the emergency room; the following signs or symptoms were assessed: fever (≥38°C), headache, cough, sore throat, rhinorrhea, myalgia, joint pain, dyspnea, conjunctivitis, sore back, diarrhea, asthenia, nausea, and/or vomiting. Systemic Inflammatory Response Syndrome (SIRS) was diagnosed according to the definition of the American College of Chest Physicians/Society of Critical Care Medicine Consensus Conference (27). None of the patients had bacterial co-infections or previous influenza vaccination. Afterwards, blood specimens from each subject were collected in silicone and EDTA-coated tubes (BD Vacutainer, Franklin Lakes, NJ). Serum samples were obtained and stored at −70°C and EDTA samples were processed immediately. Due to logistic problems, in some cases we were unable to obtain serum and blood cells from the same subject for all immune evaluations.

### Determination of Anti-Influenza Titers

The titer of anti-hemagglutinin antibodies was determined by hemagglutination inhibition test. Serum samples were heated at 56°C for 30 min and serially diluted in PBS-BSA, pH = 6.9. Fifty μL of serum dilutions were incubated for 30 min at 37°C with an equal volume of pre-diluted virus (influenza A/Mexico/4482/2009, provided by InDRE) containing 8 HA units. After incubation, titers were measured by inverse dilution where 100% of erythrocytes agglutinated. Titers of 1:40 or above were considered positive 28, 29.

### Cytokine and Chemokine Quantification

Serum cytokine (IL-2, IL-4, IL-6, IL-10, TNF-α, IFN-γ and IL-17A) and chemokine (CXCL8, CXCL9, CCL2 and CXCL10) concentrations were determined using a cytometric bead array (CBA) kit (BD PharMingen, San Diego, CA), according to the manufacturer's instructions. Log-transformed data were used to construct standard curves that were fitted to 10 discrete points using a 4-parameter logistic model. The concentrations in the test samples were calculated interpolating from their corresponding standard curves. Data analysis was performed using the GraphPad Prism software (GraphPad Software, San Diego, CA).

### Peripheral Leukocyte Surface Marker Assessments

**Supplementary Figure 1:**
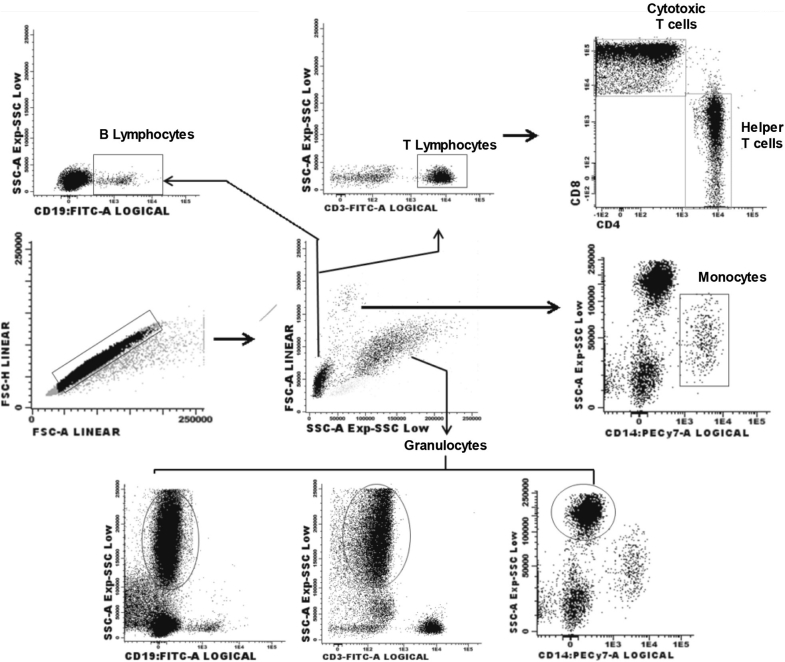


**Supplementary Figure 2:**
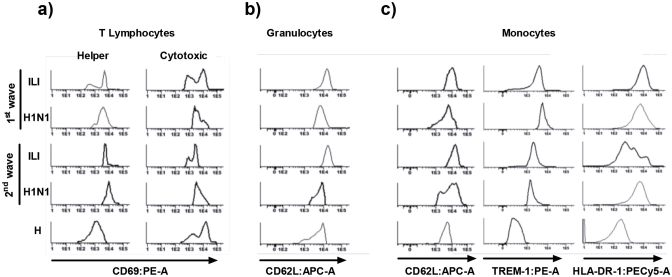


Whole blood samples were evaluated for: a) autofluorescence; b) six-color antibody-conjugated cocktail, which included anti-CD19/FITC (Clone: HIB19, BD Bioscience), anti-TREM-1/PE (Clone: 193015, R&D Systems), anti-CD86/PE-Cy5 (Clone: FUN-1, BD Bioscience), anti-CD14/PE-Cy7 (Clone: M5E2, BD Bioscience), anti-CD62L/APC (Clone: Dreg-56, Invitrogen) and anti-HLA-DR/APC-Cy7 (Clone: M5E2, BD Bioscience); or c) five-color cocktail, which included anti-CD3/FITC (Clone: HIT3, BD Bioscience), anti-CD69/PE (Clone: HIB19, BD Bioscience), anti-CD4/PE-Cy5 (Clone: RPA-T4, BD Bioscience), anti-CD8/APC (Clone: 3B5, Invitrogen) and anti-CD19/PerCP (Clone: SJ25-C1, Caltag). Appropriate isotype controls were also used. After 15 min in the dark at room temperature (RT), 500 μL of FACS Lysing Solution (Becton-Dickinson, CA) were added, and incubated for 10 min at RT. The cell suspensions were washed once with a 1 mL fraction of phosphate-buffered saline by centrifugation at 900 × g for 5 min at RT. Ten thousand single leukocytes were acquired using FACSDiva 6.1.3 software in a FACSAria I flow cytometer (BD Biosciences). Final analysis was performed using the Infinicyt Analytical Software (Cytognos). The analysis algorithms to identify and characterize leukocytes are presented in Supplementary Figure 1. Percentages of CD69, CD62L, TREM-1 and HLA-DR positive cells were determined in: CD3^+^CD4^+^ (helper T lymphocytes), CD3^+^CD8^+^ (cytotoxic T lymphocytes), CD19^+^ (B-lymphocytes), CD14^+^ (monocytes) or granulocyte-gated cells (defined as FSC^high^SSC^high^ as routinely analyzed in clinical hematocytometers) (30). Additionally, granulocytes were CD3^−^CD19^−^CD14^−^ (31). For each population, the relative expression of each marker was determined by Mean Fluorescence Intensity (MFI). Representative histograms are presented in Supplementary Figure 2.

### Statistical Analysis

To know if medians were different among groups, Kruskal-Wallis test and Dunn's multiple comparison post-test were calculated; *p* <0.05 was considered statistically significant. All statistical analyses and graphics were performed with Prism 5 software (Graphpad Software, La Jolla, CA).

## Results

### Demographic and Clinical Characteristics

Demographic and clinical characteristics of the patients for the 1^st^ and 2^nd^ influenza waves are summarized in Table 1, Table 2. Based on gender distribution, no significant differences were detected among the groups. However, we found that ILI patients were significantly older than H1N1 patients in the 2^nd^ wave (*p* <0.05). Major influenza signs and symptoms, underlying conditions, and fatal outcomes were similar among groups. In contrast, the SIRS rate was higher in the 1^st^ wave (60% for ILI and 90% for H1N1) compared to the 2^nd^ wave (15% for ILI and 17% for H1N1). Both ILI and H1N1 patients from the 1^st^ wave were negative to anti-A(H1N1)pdm2009 antibodies, showing titers <40; on the other hand, seven patients (five ILI and two H1N1) in the 2^nd^ wave were positive for anti-A(H1N1)pdm2009 with titers >40 (Table 2).

No hypercytokinemia but differential cytokine/chemokine concentrations between waves are described. Table 3 summarizes the cytokine and chemokine determinations. No significant differences were found for IL-2, TNF-α, IFN-γ, IL-17A, CXCL10 and CXCL9 levels among groups. Additionally, outlier values had no correlation with any ILI or H1N1 group, disease severity or fatal outcome. IL-6 concentration in ILI and H1N1 groups was higher than in H group for the 1^st^ and 2^nd^ waves (*p* <0.05). In contrast, CXCL8 was significantly lower in ILI and H1N1 compared to the H group in the 1^st^ wave (*p* <0.05), and still lower in the 2^nd^ wave; however, they did not reach statistical significance. Finally, IL-4 concentration in the H1N1 group was higher than in the ILI group (*p* <0.05); IL-10 concentration in the H1N1 was elevated in contrast with the H group (*p* <0.05), and IL-4 and IL-10 concentrations in the H1N1 group were higher in the 2^nd^ wave compared to the 1^st^ wave.

### Leucocyte Activation Is More Evident During the 2^nd^ Pandemic Wave

**Figure 1:**
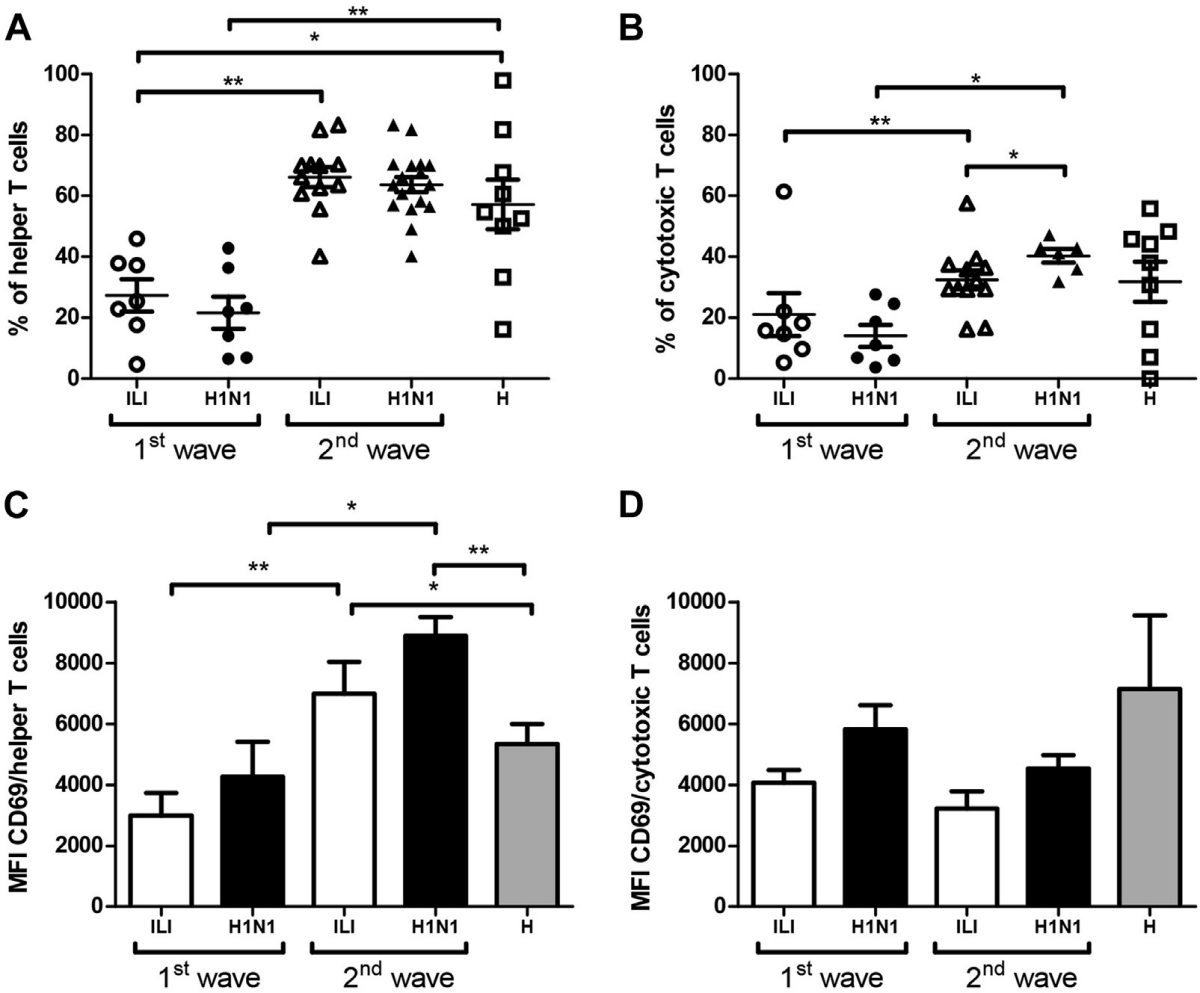
T cell phenotype in the ILI, H1N1 and H groups from the 1^st^ and 2^nd^ pandemic waves. Peripheral blood leukocytes were immunostained with CD3-, CD4-, CD8- and CD69-specific antibodies and analyzed with flow cytometry. The CD69 percentages and relative expression levels in helper (A,C) and cytotoxic (B,D) T cells are shown as mean and standard deviation. A Kruskal-Wallis test, which was followed by a Dunn's multiple comparison test, was performed. *p <0.05, **p <0.01.

No significant differences in the peripheral percentages of granulocytes, monocytes or lymphocytes were observed among groups (Supplementary Table 1). In contrast, percentage of helper T cells in the ILI and H1N1 groups was significantly lower in the 1^st^ wave than in the 2^nd^ wave (*p* <0.01); helper T cells in the ILI and H1N1groups were also significantly lower than in the H group (Supplementary Table 1 and Figure 1A, *p* <0.05 and *p* <0.01, respectively). Similar results were observed for cytotoxic T cells in ILI and H1N1 groups where the percentage was lower in the 1^st^ wave than in the 2^nd^ wave (Supplementary Table 1 and Figure 1B, *p* <0.01 and *p* <0.05).

**Figure 2:**
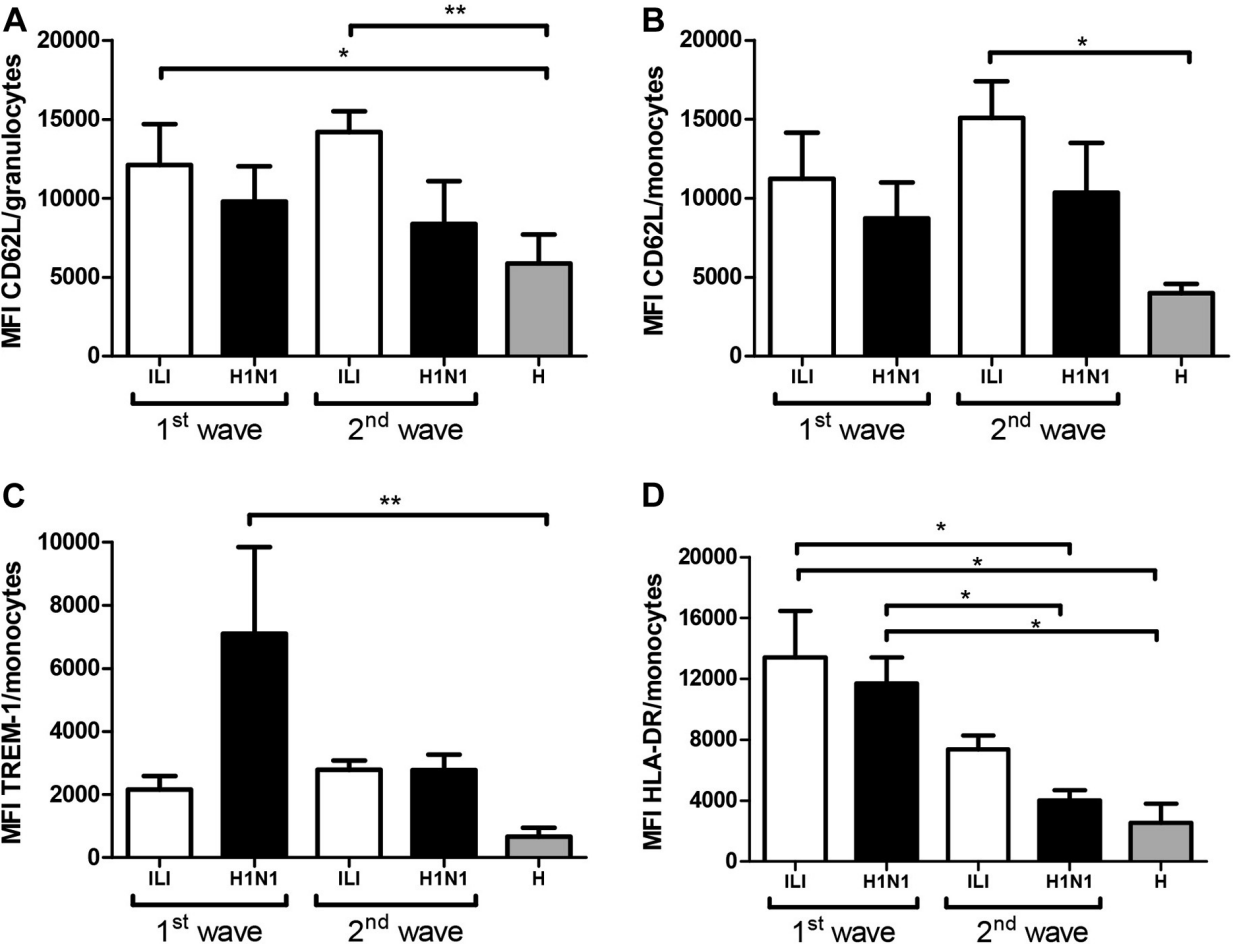
Monocyte and granulocyte phenotypes in the 1^st^ and 2^nd^ pandemic waves for the ILI, H1N1 and H groups. Peripheral blood leukocytes were immunostained with CD14-, CD62L-, TREM-1- and HLA-DR-specific antibodies and analyzed with flow cytometry. The relative neutrophil CD62L (A), monocyte CD62L (B), monocyte TREM-1 (C) and monocyte HLA-DR (D) expression levels are shown as mean and standard deviation. Kruskal-Wallis test followed by Dunn's multiple comparison test was performed. *p <0.05, **p <0.01.

Finally, we observed that CD69 expression on helper T cells in the ILI and H1N1 groups was lower in the 1^st^ wave compared to the 2^nd^ wave (*p* <0.01 and *p* <0.05, respectively); however, for the 2^nd^ wave, CD69 expression on helper T cells was higher in the ILI and H1N1 groups in comparison with the H group (Figure 1C; *p* <0.05 and *p* <0.01, respectively). We did not observe statistical differences for the expression of CD69 in cytotoxic T cells (Figure 1D). CD62L expression in granulocytes and monocytes in the ILI group was higher than in the H group at the 2^nd^ wave (Figure 2A–B granulocytes: *p* <0.01; monocytes: *p* <0.05). Unlike CD62L expression, TREM-1 expression in monocytes in the 1^st^ wave was higher in the H1N1 group than in the H group (Figure 2C, *p* <0.01). Additionally, HLA-DR expression in monocytes in both groups was higher in the 1^st^ wave than in the 2^nd^ wave, and both waves displayed higher expression in comparison with the H group (Figure 2D, *p* <0.05).

## Discussion

Influenza A(H1N1)pdm09 virus has shown different rates of morbidity and mortality among different countries, seasons and waves 26, 32, 33, reaching less mortality than the 1918 pandemic virus (0.01–0.06 vs. 2–3%) (34). However, the influenza A(H1N1)pdm09 virus had a higher lethality rate in young adults compared to neonates and elderly persons, but similar lethality in patients immunocompromised or affected by comorbidities during the 2009 outbreak (35). We did not find any age-associated or clinical differences between the waves that could explain a different immunological response against the pandemic virus (Table 1, Table 2). Moreover, we observed that the comorbidity and clinical diagnosis was not useful to differentiate between the ILI and the H1N1 groups as reported previously (36). However, we observed a lack of antibody titers >40 in the 1^st^ wave patients, supporting the hypothesis for a faint immunological response against influenza A(H1N1)pdm09 virus in the first wave. In contrast, seven patients (five ILI and two H1N1) expressed titers >40 during the 2^nd^ wave, suggesting an elevated immune response during the 2^nd^ wave. Seroprevalence analysis in Mexico showed that 39% of the population was positive after the 2^nd^ wave, with significantly higher titers in subjects <20 years of age (49.5%); interestingly one third of the seropositive subjects were asymptomatic (36). This could result from vaccination programs leading to homologous reinfections (37), suggesting a wide spread of wild type pandemic virus resulting in inter-pandemic influenza exposure, thus providing short-lived protection (38). Mathematical models have noted that early vaccination programs lead to limitation for a 2^nd^ wave; however, despite an early vaccination program in Mexico a 2^nd^ wave developed, suggesting a greater virulence of the influenza A(H1N1)pdm09 virus.

Influenza H1N1 1918 and H5N1 avian viruses are known for eliciting a hyper-immune response that included high circulating cytokine and chemokine concentrations (39). In addition, studies in patients with severe illness during the 2006–2007 seasonal influenza, showed a high concentration of IL-6 (20) and IL-8 (21); in contrast, *in vitro* studies showed the influenza virus as a poor inducer of pro-inflammatory cytokines in macrophages and dendritic cells (40). Our study reveals that IL-6 was significantly elevated (>10-fold above controls) in both pandemic waves (Table 3), suggesting a better infection control because high concentrations of IL-6 have proven to confer protective effects against influenza (41). In contrast with other reports associating IL-6 augmentation with a poor outcome (42) or SIRS (43), recent reports show that IL-6 is not a definitive biomarker to determine systemic inflammation (42). Moreover, we observed that IL-10 concentration during the 2^nd^ wave was higher in the H1N1 group than in the H group. High concentrations of IL-10 could resolve influenza infection by promoting virus clearance and autoregulation 44, 45. Probably in the 1^st^ wave IL-10 was diminished in young patients and increased in elderly patients (46).

T cell lymphopenia was associated with influenza A(H1N1)pdm09 infections 47, 48, probably as a result of differential lymphocyte migration into the lungs 49, 50. We observed T cell lymphopenia in patients from the 1^st^ but not the 2^nd^ wave. Helper T cells showed an increase in early activation markers such as CD69 only during the 2^nd^ wave (Figure 1). This could be related to differential migration patterns to lymph nodes and lungs from naive and, predominantly, memory T lymphocytes 51, 52. The higher proportion of circulating CD69^+^ CD4 T cells during the 2^nd^, but not the 1^st^ wave, could be explained by memory induced by group immunizations.

Neutrophils, the major component of granulocytes, could contribute to lung damage; however, reduced numbers or impaired function of these cells may lead to severe influenza disease (53) with a high mortality rate 54, 55. Because granulocytes could enter the lungs by using CD62L (56), we tested its expression in peripheral blood. An augmented CD62L expression in granulocytes was observed for both waves in all patients compared with the H group. Although CD62L is diminished during some infections and influenza virus *in vitro* induces CD62L shedding on neutrophils (57), it has also been reported that CD62L is upregulated in circulating leukocytes early after injury (58). Accordingly, CD62L is overexpressed in human proinflammatory neutrophils exposed to IFN-γ 59, 60, which is elevated during acute stages of illness in influenza infection (61). In addition, expression of TREM-1 and HLA-DR was also evaluated in monocytes. Interestingly, we observed increased levels in TREM-1 only during the 1^st^ wave, suggesting a wave-dependent differential migration in H1N1 patients; therefore, we propose that the expression of both CD62L and TREM-1 could be useful in the study of waves in influenza infection.

Considering all statistical differences observed in the ILI and H1N1 groups compared with the H group, our results showed greater immunological activity in the 2^nd^ wave compared with the 1^st^ wave of influenza during the 2009 outbreak; accordingly the mortality rates were greater during the 2^nd^ wave (62). Our study suggests that some differential leukocyte phenotypes such as CD69 for helper T cells, and CD62L, TREM-1 and HLA-DR for myeloid cells could explain this epidemiological observation. Our study has some limitations associated with: a) a relatively small number of observations and sizeable SD for most parameters; b) serial samples were not collected; therefore, a peak time point could have been missed; and c) signs and symptoms from patients were not stratified, just registered as present or absent.

In conclusion, to our knowledge this is the first report that describes differences in the immune response during pandemic periods, with prominent activation during the 2^nd^ pandemic wave, which was more likely explained by the immune response than by underlying diseases or age. Further investigation is needed to fully understand the underlying mechanisms driving the wave-like behavior of immune responses against pandemic influenza virus so that more focused therapeutic strategies can be designed.

## Figures and Tables

**Table 1:** Epidemiological data

	1^st^ wave (*n* = 19)		2^nd^ wave (*n* = 43)		H (*n* = 12)
ILI (*n* = 10)	H1N1 (*n* = 9)	ILI (*n* = 20)	H1N1 (*n* = 23)
Gender					
Female	4	4	12	10	7
Male	6	5	8	13	5
Age (years)					
Media	41	38.2	48^a^^∗∗^/b^∗^	30.3^b^^∗^	30.8^a^^∗∗^
Max	49	78	76	59	64
Min	15	15	18	16	22

1^st^ wave: April–May 2009; 2^nd^ wave: October 2009–February 2010.

^∗^*p* <0.05.

^∗∗^*p* <0.01.

a ILI or H1N1 (2^nd^ wave) vs. H.

b ILI vs. H1N1 at the same wave; Kruskal-Wallis test with Dunn's multiple comparison post-test.

**Table 2:** Clinical data

	1^st^ wave		2^nd^ wave	
ILI (*n* = 10)	H1N1 (*n* = 9)	ILI (*n* = 20)	H1N1 (*n* = 23)
Major signs and symptoms				
Fever (>38°C)	10/10	8/9	20/20	23/23
Headache	8/10	9/9	19/20	21/23
Cough	8/10	9/9	20/20	23/23
Throat pain	4/10	6/9	4/20	6/23
Diarrhea	3/10	0/9	1/20	1/23
Days of evolution at diagnosis	3.7 ± 2.8	5.7 ± 3.6	4.2 ± 4.5	4.5 ± 5.9
SIRS	6/10^a∗^	8/9^b∗^	3/20^a∗^	4/23^b∗^
Underlying conditions				
Tobacco consumption	3/10	6/9	8/20	5/23
Diabetes	0/10	1/9	5/20	2/23
COPD	0/10	4/9	2/20	1/23
Dyslipidemia	1/10	2/9	3/20	1/23
Hypertension	0/10	4/9	7/20	2/23
Asthma	1/10	2/9	4/20	2/23
H1N1pdm2009 antibody titers (>40)	0/10	0/9	5/20	2/23
Fatal outcome	0/10	0/9	2/20	0/23

SIRS, systemic inflammatory response syndrome; COPD, chronic obstructive pulmonary disease; 1^st^ wave: April–May 2009; 2^nd^ wave: October 2009–February 2010; ^a^ILI (1^st^ wave) vs. ILI (2^nd^ wave); ^b^H1N1 (1^st^ wave) vs. H1N1 (2^nd^ wave); Kruskal-Wallis test with Dunn's multiple comparison post-test.

^∗^*p* <0.05.

**Table 3:** Serum cytokine and chemokine levels

Cytokine/Chemokine (pg/mL)	1^st^ wave		2^nd^ wave		H (*n* = 12)
ILI (*n* = 7)	H1N1 (*n* = 9)	ILI (*n* = 18)	H1N1 (*n* = 23)
IL-2	2.9 ± 0.9	LOD	2.5 ± 0.3	2.7 ± 0.8	LOD
IL-4	6.0 ± 3.2	5.4 ± 4.6	3.9 ± 59.1^b^^∗^	14.3 ± 40.4^b^^∗^	3.9 ± 0.00
IL-6	16.3 ± 21.1	47.7 ± 85.1	58.9 ± 257.4^a^^∗^	173.4 ± 795.3^a^^∗^	2.3 ± 1.2^a^^∗^
IL-10	LOD	4.9 ± 2.5	5.5 ± 27.2	11.4 ± 20.5^a^^∗^	3.9 ± 1.3^a^^∗^
TNF-α	LOD	LOD	3.4 ± 2.6	3.3 ± 2.30	LOD
IFN-γ	LOD	LOD	5.6 ± 8.8	7.8 ± 16.2	LOD
IL-17A	LOD	LOD	LOD	19.3 ± 3.1	22.8 ± 12
CXCL10 (μg/mL)	2 ± 1.2	3.6 ± 1.4	2.4 ± 1.9	3.5 ± 1.8	2.3 ± 1.4
CCL2	111.2 ± 124.8	156.3 ± 216.0	147.3 ± 148	665.7 ± 1044.3	192.1 ± 37.2
CXCL9 (μg/mL)	0.9 ± 0.5	1.6 ± 1.6	2.5 ± 1.9	2.9 ± 1.8	1.2 ± 0.9
CXCL8	26.3 ± 31^a^^∗/^c^∗^	35.8 ± 14.87^a^^∗/^d^∗^	236.4 ± 349^c^^∗^	301.7 ± 529.5^d^^∗^	580.7 ± 766.4^a^^∗^

Limits of detection (pg/mL): 2.6 (IL-2); 4.9 (IL-4); 2.4 (IL-6); 4.5 (IL-10); 3.8 (TNF-α); 3.7 (IFN-γ); 18.9 (IL-17A); 2.8 (CXCL10); 2.7 (CCL2); 2.5 (CXCL9); 0.2 (CXCL8); 1^st^ wave: April–May 2009; 2^nd^ wave: October 2009–February 2010; LOD: lower limit of detection. Mean ± SD.

Kruskal-Wallis test with Dunn's multiple comparison post-test; ^∗^*p* <0.05.

a ILI or H1N1 (1^st^ wave) vs. H.

b ILI vs. H1N1 at the same wave.

c ILI (1^st^ wave) vs. ILI (2^nd^ wave).

d H1N1 (1^st^ wave) vs. H1N1 (2^nd^ wave).

## References

[1] Curfman G.D., Morrissey S., Malina D. (2009). Health care reform 2009 at healthcarereform.nejm.org. N Engl J Med.

[2] Anon. WHO Pandemic (H1N1) 2009 2012.

[3] Ormsby C.E., de la Rosa-Zamboni D., Vazquez-Perez J. (2011). Severe 2009 pandemic influenza A (H1N1) infection and increased mortality in patients with late and advanced HIV disease. AIDS.

[4] Paulo A.C., Correia-Neves M., Domingos T. (2010). Influenza infectious dose may explain the high mortality of the second and third wave of 1918–1919 influenza pandemic. PLoS One.

[5] Guan Y., Poon L.L., Cheung C.Y. (2004). H5N1 influenza: a protean pandemic threat. Proc Natl Acad Sci USA.

[6] Loo Y.M., Gale M. (2007). Influenza: fatal immunity and the 1918 virus. Nature.

[7] Osterholm M.T. (2005). Preparing for the next pandemic. N Engl J Med.

[8] Lagace-Wiens P.R., Rubinstein E., Gumel A. (2010). Influenza epidemiology–past, present, and future. Crit Care Med.

[9] Bennink J.R., Doherty P.C. (1981). The response to H-2-different virus-infected cells is mediated by long-lived T lymphocytes and is diminished by prior virus priming in a syngeneic environment. Cell Immunol.

[10] Mummert A., Weiss H., Long L.P. (2013). A perspective on multiple waves of influenza pandemics. PLoS One.

[11] Elderfield R.A., Watson S.J., Godlee A. (2014). Accumulation of human-adapting mutations during circulation of A(H1N1)pdm09 influenza virus in humans in the United Kingdom. J Virol.

[12] Hsieh Y.H., Cheng K.F., Wu T.N. (2011). Transmissibility and temporal changes of 2009 pH1N1 pandemic during summer and fall/winter waves. BMC Infect Dis.

[13] Nelson M.I., Tan Y., Ghedin E. (2011). Phylogeography of the spring and fall waves of the H1N1/09 pandemic influenza virus in the United States. J Virol.

[14] Bolton K.J., McCaw J.M., McVernon J. (2014). The influence of changing host immunity on 1918–19 pandemic dynamics. Epidemics.

[15] Dorigatti I., Cauchemez S., Ferguson N.M. (2013). Increased transmissibility explains the third wave of infection by the 2009 H1N1 pandemic virus in England. Proc Natl Acad Sci U S A.

[16] Deng J.C. (2013). Viral-bacterial interactions-therapeutic implications. Influenza Other Respir Viruses.

[17] Pascalis H., Temmam S., Turpin M. (2012). Intense co-circulation of non-influenza respiratory viruses during the first wave of pandemic influenza pH1N1/2009: a cohort study in Reunion Island. PLoS One.

[18] Matrajt L., Longini I.M. (2012). Critical immune and vaccination thresholds for determining multiple influenza epidemic waves. Epidemics.

[19] Camacho A., Ballesteros S., Graham A.L. (2011). Explaining rapid reinfections in multiple-wave influenza outbreaks: Tristan da Cunha 1971 epidemic as a case study. Proc Biol Sci.

[20] Heltzer M.L., Coffin S.E., Maurer K. (2009). Immune dysregulation in severe influenza. J Leukoc Biol.

[21] Lee N., Wong C.K., Chan P.K. (2011). Cytokine response patterns in severe pandemic 2009 H1N1 and seasonal influenza among hospitalized adults. PLoS One.

[22] Cerbulo-Vazquez A., Figueroa-Damian R., Arriaga-Pizano L.A. (2014). Pregnant women infected with pandemic H1N1pdm2009 influenza virus displayed overproduction of peripheral blood CD69+ lymphocytes and increased levels of serum cytokines. PLoS One.

[23] Ferat-Osorio E., Esquivel-Callejas N., Wong-Baeza I. (2008). The increased expression of TREM-1 on monocytes is associated with infectious and noninfectious inflammatory processes. J Surg Res.

[24] Pelikan Z. (2014). Expression of surface markers on the blood cells during the delayed asthmatic response to allergen challenge. Allergy Rhinol (Providence).

[25] Echevarria-Zuno S., Mejia-Arangure J.M., Mar-Obeso A.J. (2009). Infection and death from influenza A H1N1 virus in Mexico: a retrospective analysis. Lancet.

[26] CDC Protocol of Realtime RT-PCR for Influenza A(H1N1) Centers for Disease Control and Prevention: Atlanta, GA; 2009. http://www.who.int/csr/resources/publications/swineflu/CDCRealtimeRTPCR_SwineH1Assay-2009_20090430.pdf

[27] Bone R.C., Balk R.A., Cerra F.B. (1992). Definitions for sepsis and organ failure and guidelines for the use of innovative therapies in sepsis. The ACCP/SCCM Consensus Conference Committee. American College of Chest Physicians/Society of Critical Care Medicine. Chest.

[28] (CDC) CfDCaP (2009). Serum cross-reactive antibody response to a novel influenza A (H1N1) virus after vaccination with seasonal influenza vaccine. MMWR Morb Mortal Wkly Rep.

[29] Potter C.W., Oxford J.S. (1979). Determinants of immunity to influenza infection in man. Br Med Bull.

[30] Taieb A. Part Four: Basic concepts in hematology. In: Services AUP, ed. Understanding & Interpreting Hematological Investigations. 1st ed. Aberdeen University Press Services: United Kingdom 2009. pp. 89.

[31] Gopinath R., Nutman T.B. (1997). Identification of eosinophils in lysed whole blood using side scatter and CD16 negativity. Cytometry.

[32] Lopez-Cuadrado T., de Mateo S., Jimenez-Jorge S. (2012). Influenza-related mortality in Spain, 1999–2005. Gac Sanit.

[33] Viboud C., Boelle P.Y., Pakdaman K. (2004). Influenza epidemics in the United States, France, and Australia, 1972–1997. Emerg Infect Dis.

[34] Maeda N., Uede T. (2010). Swine-origin influenza-virus-induced acute lung injury: Novel or classical pathogenesis?. World J Biol Chem.

[35] Aldridge J.R., Moseley C.E., Boltz D.A. (2009). TNF/iNOS-producing dendritic cells are the necessary evil of lethal influenza virus infection. Proc Natl Acad Sci U S A.

[36] Elizondo-Montemayor L., Hernandez-Torre M., Ugalde-Casas P.A. (2012). Clinical and epidemiological features of 2009 pandemic H1N1 influenza differ slightly according to seroprevalence status during the second wave in the general population in Mexico. Respir Care.

[37] Camacho A., Cazelles B. (2013). Does homologous reinfection drive multiple-wave influenza outbreaks? Accounting for immunodynamics in epidemiological models. Epidemics.

[38] Mathews J.D., McCaw C.T., McVernon J. (2007). A biological model for influenza transmission: pandemic planning implications of asymptomatic infection and immunity. PLoS One.

[39] Baskin C.R., Bielefeldt-Ohmann H., Tumpey T.M. (2009). Early and sustained innate immune response defines pathology and death in nonhuman primates infected by highly pathogenic influenza virus. Proc Natl Acad Sci U S A.

[40] Osterlund P., Pirhonen J., Ikonen N. (2010). Pandemic H1N1 2009 influenza A virus induces weak cytokine responses in human macrophages and dendritic cells and is highly sensitive to the antiviral actions of interferons. J Virol.

[41] Lauder S.N., Jones E., Smart K. (2013). Interleukin-6 limits influenza-induced inflammation and protects against fatal lung pathology. Eur J Immunol.

[42] Paquette S.G., Banner D., Zhao Z. (2012). Interleukin-6 is a potential biomarker for severe pandemic H1N1 influenza A infection. PLoS One.

[43] Giannoudis P.V., Harwood P.J., Loughenbury P. (2008). Correlation between IL-6 levels and the systemic inflammatory response score: can an IL-6 cutoff predict a SIRS state?. J Trauma.

[44] Bot A., Holz A., Christen U. (2000). Local IL-4 expression in the lung reduces pulmonary influenza-virus-specific secondary cytotoxic T cell responses. Virology.

[45] Sun J., Madan R., Karp C.L. (2009). Effector T cells control lung inflammation during acute influenza virus infection by producing IL-10. Nature Med.

[46] Mohanty S., Joshi S.R., Ueda I. (2015). Prolonged proinflammatory cytokine production in monocytes modulated by interleukin 10 after influenza vaccination in older adults. J Infect Dis.

[47] Cui W., Zhao H., Lu X. (2010). Factors associated with death in hospitalized pneumonia patients with 2009 H1N1 influenza in Shenyang, China. BMC Infect Dis.

[48] Hagau N., Slavcovici A., Gonganau D.N. (2010). Clinical aspects and cytokine response in severe H1N1 influenza A virus infection. Crit Care.

[49] Mackenzie C.D., Taylor P.M., Askonas B.A. (1989). Rapid recovery of lung histology correlates with clearance of influenza virus by specific CD8+ cytotoxic T cells. Immunology.

[50] Iezzi G., Scheidegger D., Lanzavecchia A. (2001). Migration and function of antigen-primed nonpolarized T lymphocytes in vivo. J Exp Med.

[51] Sathaliyawala T., Kubota M., Yudanin N. (2013). Distribution and compartmentalization of human circulating and tissue-resident memory T cell subsets. Immunity.

[52] Turner S.J., Cross R., Xie W. (2001). Concurrent naive and memory CD8(+) T cell responses to an influenza A virus. J Immunol.

[53] Brandes M., Klauschen F., Kuchen S. (2013). A systems analysis identifies a feedforward inflammatory circuit leading to lethal influenza infection. Cell.

[54] Tate M.D., Ioannidis L.J., Croker B. (2011). The role of neutrophils during mild and severe influenza virus infections of mice. PLoS One.

[55] Tate M.D., Deng Y.M., Jones J.E. (2009). Neutrophils ameliorate lung injury and the development of severe disease during influenza infection. J Immunol.

[56] Wedepohl S., Beceren-Braun F., Riese S. (2012). L-selectin–a dynamic regulator of leukocyte migration. Eur J Cell Biol.

[57] Hartshorn K.L., White M.R. (1999). Influenza A virus up-regulates neutrophil adhesion molecules and adhesion to biological surfaces. J Leukoc Biol.

[58] De Martinis M., Modesti M., Ginaldi L. (2004). Phenotypic and functional changes of circulating monocytes and polymorphonuclear leucocytes from elderly persons. Immunol Cell Biol.

[59] Kamp V.M., Leentjens J., Pillay J. (2013). Modulation of granulocyte kinetics by GM-CSF/IFN-gamma in a human LPS rechallenge model. J Leukoc Biol.

[60] Pillay J., Kamp V.M., van Hoffen E. (2012). A subset of neutrophils in human systemic inflammation inhibits T cell responses through Mac-1. J Clin Invest.

[61] Khatri M., Dwivedi V., Krakowka S. (2010). Swine influenza H1N1 virus induces acute inflammatory immune responses in pig lungs: a potential animal model for human H1N1 influenza virus. J Virol.

[62] Secretaria de Salud M. SINAIS/SINAVE/DGE/SALUD/Perfil epidemiológico de la pandemia de Influenza A (H1N1) 2009 en México 2011.

