# Supplementary material for: Differential Immune Profiles in Two Pandemic Influenza A(H1N1)pdm09 Virus Waves at Pandemic Epicenter
Source: Arch Med Res. 2015 Nov;46(8):651–8. doi: 10.1016/j.arcmed.2015.12.003 (PMC4914610; doi:10.1016/j.arcmed.2015.12.003)
Supplement: Supplementary Table 1 and Legends for Supplementary Figures 1 and 2 [file mmc1.docx]

Supplementary Table 1. Percentage of leukocytes in whole blood

| **Leukocytes (%)** | **1^st^ wave (*n* = 19)** | | **2^nd^ wave (*n* = 43)** | | **H (*n* = 12)** |
| --- | --- | --- | --- | --- | --- |
|  | **ILI (*n* = 10)** | **H1N1 (*n* = 9)** | **ILI (*n* = 20)** | **H1N1 (*n* =23)** |  |
| **From total** |  |  |  |  |  |
| Granulocytes | 48.7±14.3 | 53.2±18.3 | 60.4±12.0 | 47.1±14.1 | 44.3±9.6 |
| Monocytes | 4.5±2.1 | 5.4±3.1 | 7.1±3.8 | 5.6±2.6 | 8.3±2.1 |
| B cells | 5.0±2.7 | 8.2±5.0 | 5.7±4.1 | 6.4±4.2 | 12.6±15.1 |
| T cells | 27.7±13.7 | 26.4±10.9 | 17.6±11.2 | 24.0±13.1 | 32.2±8.3 |
| **From T cells** | | |  | | |
| Helper | 27.3±14.1^a*/b**^ | 21.6±14.0^a**^ | 66.2±11.4^b**^ | 58.6±6.4 | 57.2±24.3^a*/a**^ |
| Cytotoxic | 21.0±18.6^b**^ | 14.0±9.5^c*^ | 32.4±10.8^b**^^/d*^ | 40.3±5.5 ^c*/d*^ | 31.8±19.7 |

1^st^ wave: April-May 2009; 2^nd^ wave: October 2009–February 2010.

^a^ILI or H1N1 (1^st^ wave) vs. H.

^b^ILI (1^st^ wave) vs. ILI (2^nd^ wave).

^c^H1N1 (1^st^ wave) vs. H1N1 (2^nd^ wave).

^d^ILI vs H1N1 at the same wave.

Kruskal-Wallis test with Dunn’s multiple comparison post-test.

**p* <0.05; ***p* <0.01.

Figure Legend

Supplementary Figure 1. Analysis algorithm for leucocyte identification. From single events (FSC-A vs. FSC-H dot plot), major leucocyte subpopulations were selected for size (FSC) vs. granularity (SSC) pattern. From lymphocyte population, both B lymphocytes (CD19^+^), and T lymphocyte (CD3^+^), were defined, as well as helper T cells (CD3^+^CD4^+^) and cytotoxic T cells (CD3^+^CD8^+^). Monocytes are CD14^+^, whereas granulocytes were defined by heterogeneous size, high granularity (FSC^high^, SSC^high^) and CD3^-^CD14^-^CD19^-^.

Supplementary Figure 2. Representative histograms for relative expression of CD69 in helper T cells (a), CD69 in cytotoxic T cells (b), CD62L in granulocytes (c) and CD62L, TREM-1 and HLA-DR in monocytes (d).
